# Natural history of intracholecystic papillary neoplasm (ICPN): a rare case of ICPN whose natural history was closely followed by ultrasound

**DOI:** 10.1186/s12876-022-02454-z

**Published:** 2022-08-08

**Authors:** Daisuke Koike, Hiroyuki Kato, Yukio Asano, Masahiro Ito, Satoshi Arakawa, Norihiko Kawabe, Masahiro Shimura, Chihiro Hayashi, Takayuki Ochi, Kenshiro Kamio, Toki Kawai, Hironobu Yasuoka, Takahiko Higashiguchi, Akihiko Horiguchi

**Affiliations:** Department of Gastroenterological Surgery, Fujita Health University School of Medicine, Bantane Hospital, 3-6-10 Otobashi Nakagawa Ward, Nagoya, Aichi 454-8509 Japan

**Keywords:** Intracholecystic papillary neoplasm, Natural history, Sessile polyp

## Abstract

The natural history of intracholecystic papillary neoplasm (ICPN), especially the speed of growth from small benign to a carcinomatous lesion, is quite unrevealed. Here, we report an extremely rare case of ICPN, in which the papillary lesion was observed transforming from small and benign to malignant using abdominal ultrasound (AUS) over 2 years during routine health checks. A 44-year-old man underwent a routine health check-up. The initial AUS showed a small sessile polyp in the gallbladder, which enlarged slightly at the next AUS, a year later. In the third year, the polypoid lesion enlarged markedly, with a maximum diameter of 10 × 9 × 7 mm. Therefore, a laparoscopic cholecystectomy was performed. Microscopically, the 10 mm tumor had intracytoplasmic mucus, and a clear cytoplasm compatible with gastric-type features. Immunohistochemical analysis showed positive staining of atypical cells for MUC6 and PAS. These findings led to the diagnosis of ICPN with high-grade intraepithelial neoplasia of the gastric type. In conclusion, sessile polyps with rapid growth might be a crucial finding in the early stage of ICPN.

## Background

The intracholecystic papillary neoplasm (ICPN) of the gallbladder, which was established in 2010 by the WHO, and was revised in 2019 is considered a preinvasive neoplastic lesion [1, 2]. It is regarded a counterpart to pancreatic intraductal papillary mucinous neoplasm (IPMN) and intraductal papillary neoplasm of the bile duct (IPNB) in the gallbladder.

IPMN has a well-defined illness concept, and the risk of malignant transformation has been revealed in several studies [3–5]. Additionally, the indications for surgical resection are well established, with the findings of its natural history [6, 7]. Preinvasive tumor therapy can be performed to improve the prognosis of patients with pancreatic disease. Gallbladder malignancy is also one of the most important tumors with a poor prognosis [8, 9] compared to the improved prognosis of ICPN. Therefore, it is important to clarify the natural history of premalignant lesions of the gallbladder. However, its lifespan, especially the growth speed from small benign to carcinomatous lesions, has yet to be revealed. Here, we report an extremely rare case of ICPN in which the papillary lesion was observed from a small to a carcinomatous lesion for 2 years by abdominal ultrasound (AUS) during routine health checks.

## Case presentation

A 44-year-old man underwent a routine health check-up. He did not have any abdominal symptoms and his blood cell count and liver function test results were all within normal range.

**Fig. 1:**
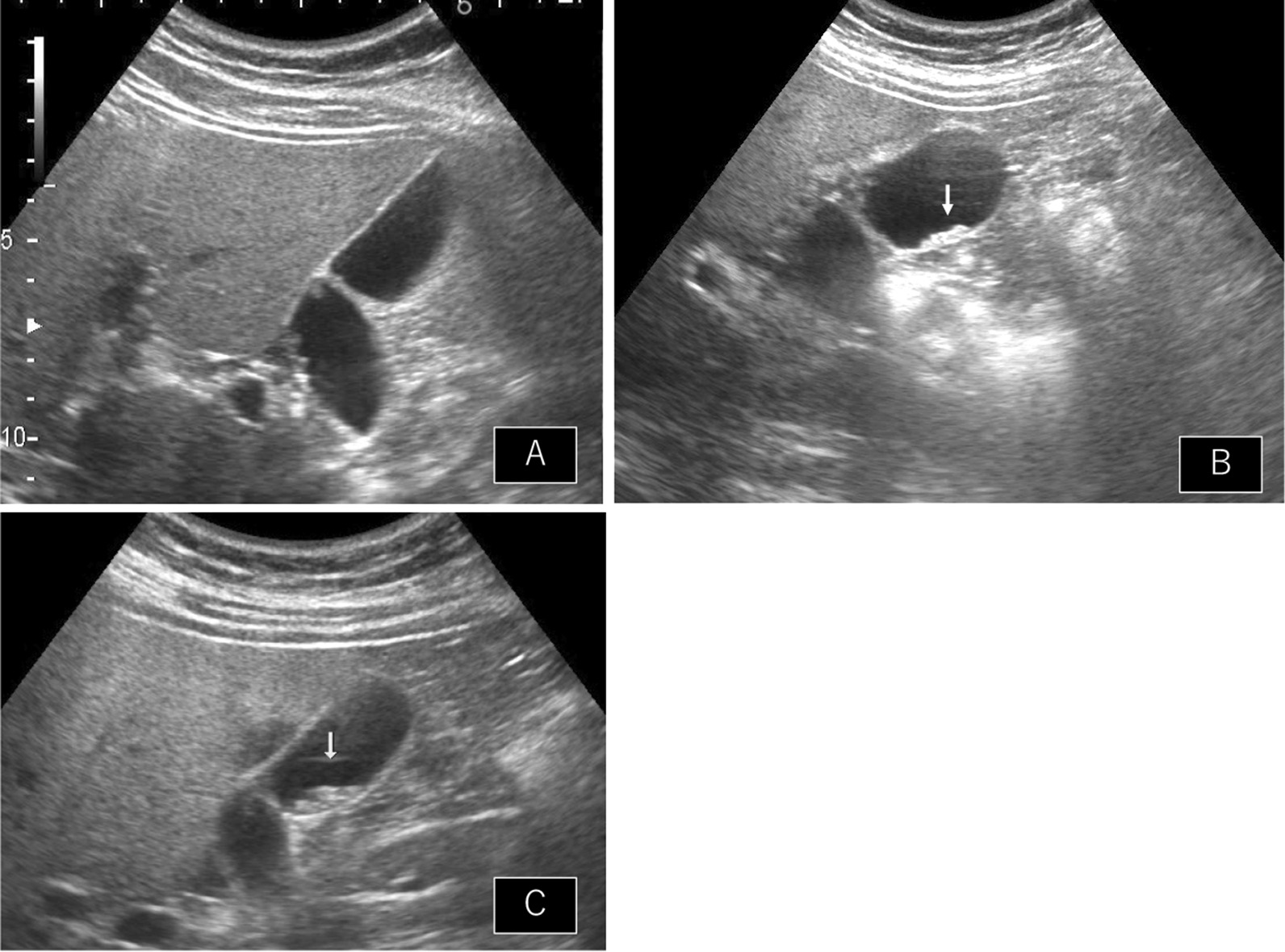
Annual change of ultrasonography’s findings. **A** Multiple polyps with maximum diameter 3 mm. **B** Sessile polyp in the gallbladder body (white arrow). **C** Sessile polyp slightly enlarged with 1 year later (white arrow)

**Fig. 2:**
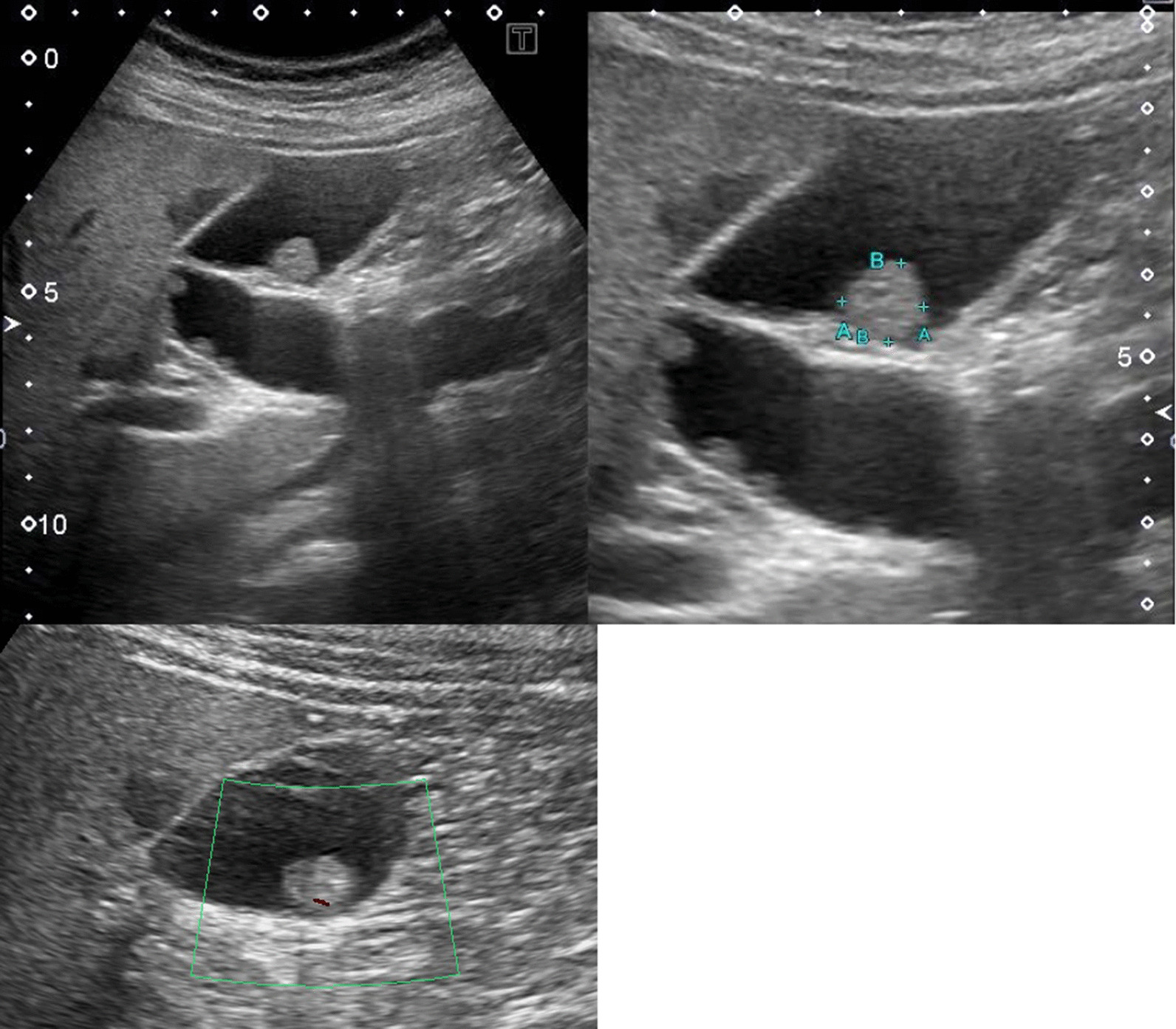
Enlarged polypoid lesion in the gallbladder wall with maximum diameter 10 × 9 × 7 mm after 2 years follow up. Doppler US found slightly blood signal in the tumor

**Fig. 3:**
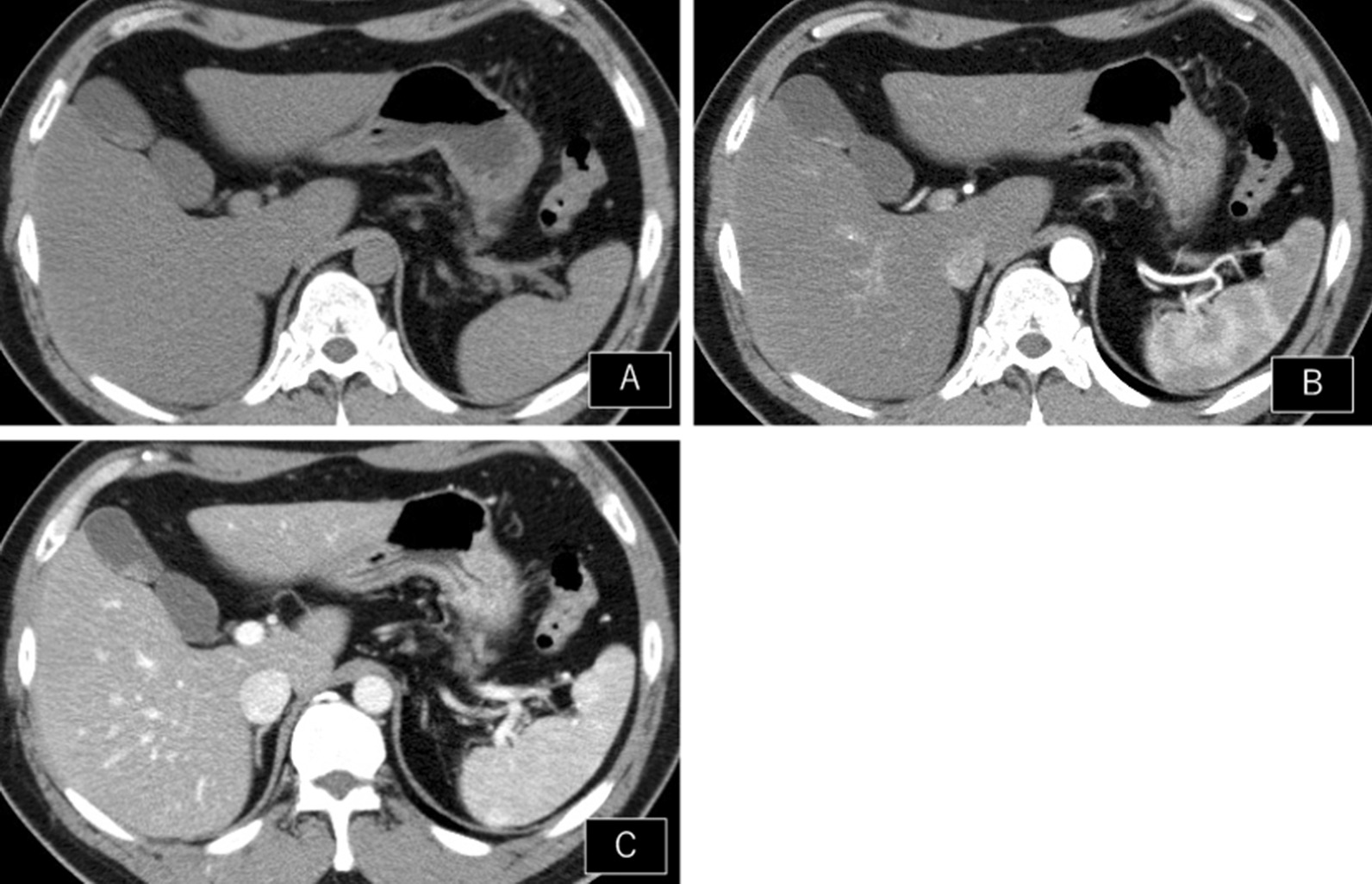
Computed tomography of the gallbladder tumor. The wall thickness shows high density in the plain (**A**) and enhancement in the early and delayed phases (**B**, **C**)

The initial AUS performed in September 2017 showed multiple polyps with a maximum diameter of 3 mm and adenomiomatosis (Fig. 1A). A sessile polyp was also found in the gallbladder (Fig. 1B). Initially, the polyps were diagnosed as cholesterol polyps. A year later, the AUS still showed a sessile polyp in the gallbladder body, which was slightly enlarged compared to that of the previous year (Fig. 1C). After another year, the polypoid lesion enlarged markedly, with a maximum diameter of 10 × 9 × 7 mm. The surface of the polyp was smooth, and the other multiple polyp lesions were enlarged (Fig. 2). Doppler US revealed a slight blood signal in the tumor. Magnetic resonance imaging (MRI) revealed that the gallbladder polyp showed high intensity T1 and low intensity T2. Magnetic resonance cholangiopancreatography (MRCP) did not reveal any anatomical anomalies of the biliary duct. Enhanced abdominal computed tomography (CT) showed the wall thickness of the gallbladder, whose density is high on the plain, and enhancement in the early and delayed phases of CT (Fig. 3). Lymphadenopathy was not identified using any imaging methods. Laboratory tests findings were unremarkable for the complete blood count or biochemical tests. Regarding tumor markers, carcinoembryonic antigen and CA19-9 levels were not elevated.

**Fig. 4:**
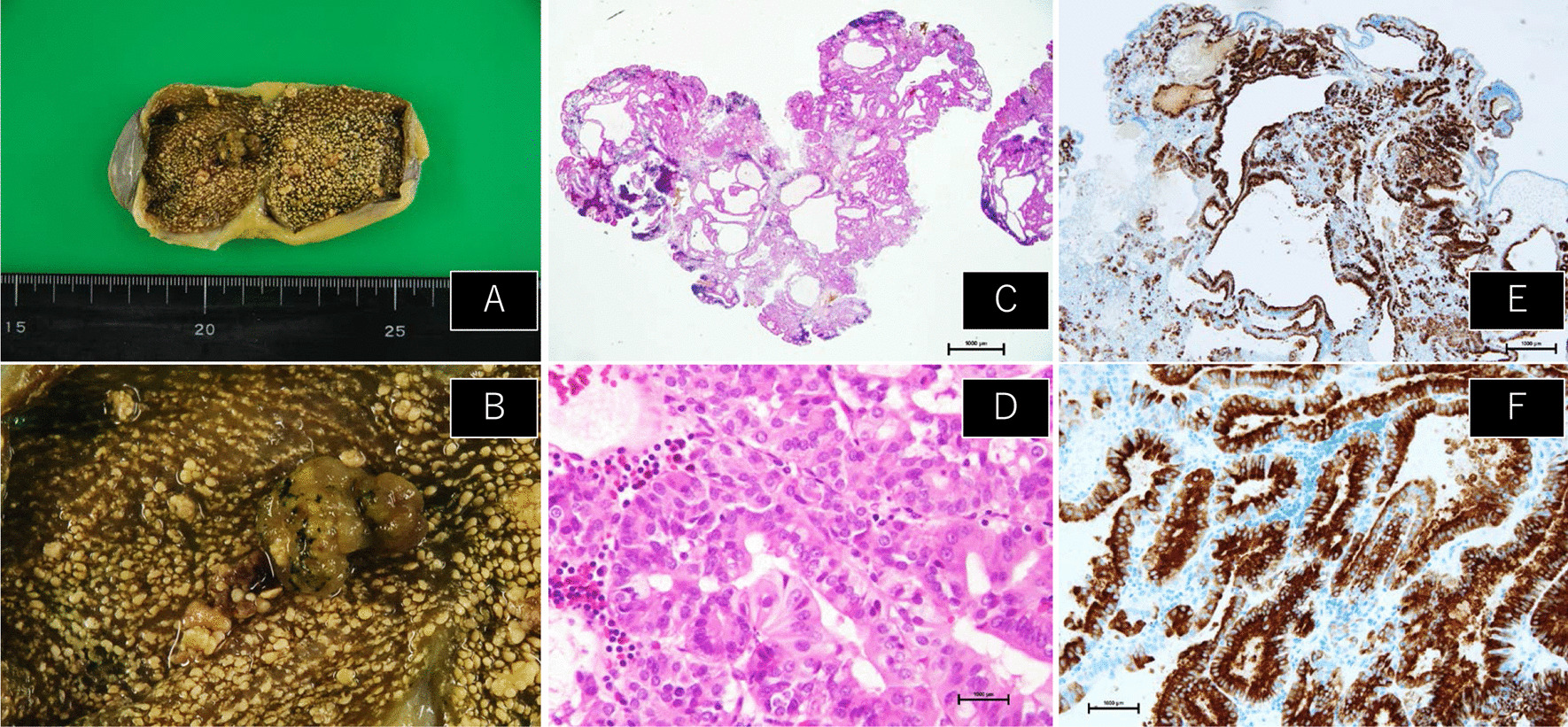
Pathological findings of resected specimen. Papillary tumor with a narrow stalk with accompanied by background obvious cholesterosis. **A**, **B** The tumor had large and small tubular structures and showed cellular pleomorphism, nuclear enlargement, and obvious nucleoli. **C**, **D** Immunohistochemistry showed strong positive staining of MUC6 (**E**) and β-catenin (**F**). (C × 2; D × 40; E × 4; F × 20) (microscope model: Olympus BX51, camera: Nikon DS-Fi3, software: NIS-Elements D)

The preoperative diagnosis was a cholesterol polyp with rapid enlargement or pyloric-type adenoma of the gallbladder. Gallbladder cancer was a differential diagnosis; however, the probability of advanced disease was estimated to be low, and we planned a laparoscopic procedure without rapid intraoperative histological diagnosis. The patient underwent laparoscopic cholecystectomy. Based on the laparoscopic findings, the gallbladder serosa was normal and there were no findings suggesting advanced cancer. The postoperative course was uneventful. Macroscopically, the resected specimen showed a 10 mm polypoid lesion along the narrow fibrovascular stalks, accompanied by obvious background cholesterolosis (Fig. 4). There was no mucus in the gallbladder. Microscopically, the lesion revealed atypical cubic and columnar cells with abundant pale cytoplasm densely grown in large and small tubular structures, with intraluminal growth of back-to-back epithelial units in the papillary architecture. Tumor cells showed cell, and nuclear enlargement as well as pleomorphism. Moreover, hyperchromasia and distinct nucleoli were observed in the tumor nuclei. Cystic and dilated glands were found in the tumor and these cells contained acidophilic-containing material. These findings are consistent with characteristics of gastric-type tumors. The tumor had no superficial spread. Immunohistochemical analysis showed positive staining of atypical cells for MUC6 and PAS. Positive staining for β-catenin was observed in both the cytoplasm and the nucleus of the tumor cells. Slight positive staining for p53 and MIB1 was also observed on atypia cells. These findings led to the diagnosis of ICPN with high-grade intraepithelial neoplasia, gastric type, according to the 5th edition of the WHO tumor classification.

## Discussion and conclusions

In the present case, we report a significant growth of ICPN from small sessile polyps to polypoid lesions with high-grade dysplasia, which could be successfully observed using AUS for two and a half years. Because the small sessile polypoid lesion was considered as benign lesion at the initial diagnosis, conservative observation was selected as the treatment option and the history of the sessile lesion was evaluated via US every year. The follow-up AUS captured the rapid enlargement of the tumor. Although clinical characteristics, etiology, and treatment options have often been discussed [10–14], the imaging findings of early stage ICPN and its natural history have rarely been reported. In literature, till date, ICPN is regarded as slow growing tumor. However, as demonstrated in the current case report, it has the potential to grow rapidly.

In general, sessile polyps in the large intestine, which are found more often in people who smoke, are known as precancerous lesions; thus, they are treated as precancerous. The cells of this type of polyp look serrated, or have a sawtooth appearance under a microscope, which gives them their name. Surveillance colonoscopies or other tests may be necessary on detection of large sessile serrated polyps. Regarding a sessile polyp lesion of the gallbladder, Ishikawa et al. [15]. reported sessile polypoid lesions of the gallbladder had more malignant potential than pedunculated polyps. Matos et al. [16]. suggested that gallbladder carcinoma is more common in patients aged > 50 years, with a single sessile lesion having a diameter of more than 10 mm. In this case, a sessile polyp was observed in the gallbladder, because its initial size was quite small. Cholecystectomy was then performed due to rapid growth for 2 years and a diameter ≥ 10 mm. In the current case, the tumor was diagnosed as gastric-type ICPN, but no invasive lesions were found in clinical and pathological findings. Gastric type ICPN suggests a high risk of malignancy, with an invasive ratio of up to 81% [17]. Therefore, we consider that the current case reflects the early stages of gastric-type ICPN. Macroscopically, the tumor developed from a sessile lesion into a papillary lesion, which then turned out to be invasive. Biliary intraepithelial neoplasm (BilIN) is the differential diagnosis of precursor lesions in the biliary tract. However, BilIN is characterised by a microscopically identifiable preinvasive neoplasm without macroscopic tumor findings and is found very rarely in the gallbladder. These characteristics were not observed in the present case. Moreover, ICPN was found in the background of cholesterolosis. Pehlivanoglu et al. [17]. reported cholesterolosis was found in 32% of ICPN cases and suggested that nonmucinous intracholecystic tubular tumors such as ICPN may arise from cholesterol polyps. However, the causality between ICPN and cholesterolosis has not yet been clarified. In the current case, cholesterolosis preceded the development of ICPN for several years, supporting the speculation that cholesterolosis is not just accompanied by ICPN, but may be a background field. Thus, taken together, we consider that a sessile lesion in cholesterolosis could suggest gastric-type ICPN.

The accumulation of genetic mutations with tumor progression was suggested in the previous case series of ICPN, and ICPN exhibited slight p53 and MIB-1 staining, reflecting its mutation in a previous case series [18]. Positive staining for β-catenin suggested activation of the Wnt/β-catenin pathway in ICPN [19]. The prognosis of ICPN is good compared to that of conventional invasive gallbladder carcinoma [20]. However, the 5-year survival rate of non-invasive ICPNs is 78%, which is worse than that of benign tumors. The early findings of ICPN are helpful for an accurate follow-up of serrated lesions in the gallbladder.

In conclusion, sessile polyps with rapid growth might be crucial findings in the early stage of ICPN, allowing us to consider the malignant potential of the gallbladder polyp. However, this is the first case study in which we followed its natural history annually, and further case accumulation is needed to clarify the clinical features of ICPN.

## Data Availability

All data generated or analysed during this study are included in the article.

## Abbreviations

ICPN: Intracholecystic papillary neoplasm
IPMN: Intraductal papillary mucinous neoplasm
IPNB: Intraductal papillary neoplasm of the bile duct
AUS: Abdominal ultrasound
MRI: Magnetic resonance imaging
CT: Computed tomography

## References

[1] Bosman FT, Carneiro F, Hruban RH, Theise ND (2010). WHO classification of tumors of the digestive system.

[2] WHO Classification of Tumors Editorial Board. WHO classification of tumors. In: Digestive system tumors, 5th edn. Lyon: International Agency for Reearch on Cancer; 2019.

[3] Kang JS, Park T, Han Y (2019). Clinical validation of the 2017 international consensus guidelines on intraductal papillary mucinous neoplasm of the pancreas. Ann Surg Treat Res.

[4] Levink I, Bruno MJ, Cahen DL (2018). Management of intraductal papillary mucinous neoplasms: controversies in guidelines and future perspectives. Curr Treat Options Gastroenterol.

[5] Min JH, Kim YK, Kim H, Cha DL, Ahn S (2020). Prognosis of resected intraductal papillary mucinous neoplasm of the pancreas: using revised 2017 international consensus guidelines. Abdom Radiol (NY).

[6] Hirono S, Yamaue H (2020). Surgical strategy for intraductal papillary mucinous neoplasms of the pancreas. Surg Today.

[7] Hwang JA, Choi SY, Lee JE (2020). Pre-operative nomogram predicting malignant potential in the patients with intraductal papillary mucinous neoplasm of the pancreas: focused on imaging features based on revised international guideline. Eur Radiol.

[8] Miyakawa S, Ishihara S, Horiguchi A, Takada T, Miyazaki M, Nagakawa T (2009). Biliary tract cancer treatment: 5,584 results from the Biliary Tract Cancer Statistics Registry from 1998 to 2004 in Japan. J Hepatobiliary Pancreat Surg.

[9] Nakanuma Y, Jang KT, Fukushima N, Furukawa T, Hong SM, Kim H, Lee KB, Zen Y, Jang JY, Kubota K (2018). A statement by the Japan-Korea expert pathologists for future clinicopathological and molecular analyses toward consensus building of intraductal papillary neoplasm of the bile duct through several opinions at the present stage. J Hepatobiliary Pancreat Sci.

[10] Mizobuchi N, Munechika J, Takeyama N (2018). Three cases of intracystic papillary neoplasm of gallbladder. Abdom Radiol (NY).

[11] Hara A, Kamata K, Takenaka M, Chikugo T, Kudo M (2019). Intracystic papillary neoplasm preoperatively diagnosed by high-quality cytology derived from endoscopic nasogallbladder drainage. Gastrointest Endosc.

[12] Jang KT, Ahn S (2016). Tumoral versus flat intraepithelial neoplasia of pancreatobiliary tract, gallbladder, and ampulla of vater. Arch Pathol Lab Med.

[13] Matthaei H, Wu J, Dal Molin M (2012). GNAS codon 201 mutations are uncommon in intraductal papillary neoplasms of the bile duct. HPB (Oxford).

[14] Meguro Y, Fukushima N, Koizumi M (2014). A case of mixed adenoneuroendocrine carcinoma of the gallbladder arising from an intracystic papillary neoplasm associated with pancreaticobiliary maljunction. Pathol Int.

[15] Ishikawa O, Ohhigashi H, Imaoka S (1989). The difference in malignancy between pedunculated and sessile polypoid lesions of the gallbladder. Am J Gastroenterol (Springer Nature).

[16] Andrén-Sandberg Å (2012). Diagnosis and management of gallbladder polyps. N Am J Med Sci.

[17] Pehlivanoglu B, Balci S, Basturk O (2021). Intracholecystic tubular non-mucinous neoplasm (ICTN) of the gallbladder: a clinicopathologically distinct, invasion-resistant entity. Virchows Arch.

[18] Fujisawa M, Matsushima M, Carreras J (2021). Whole-genome copy number and immunohistochemical analyses on surgically resected intracholecystic papillary neoplasms. Pathol Int.

[19] Akita M, Fujikura K, Ajiki T (2019). Intracholecystic papillary neoplasms are distinct from papillary gallbladder cancers: a clinicopathologic and exome-sequencing study. Am J Surg Pathol.

[20] Adsay V, Jang KT, Roa JC (2012). Intracholecystic papillary-tubular neoplasms (ICPN) of the gallbladder (neoplastic polyps, adenomas, and papillary neoplasms that are ≥1.0 cm): clinicopathologic and immunohistochemical analysis of 123 cases. Am J Surg Pathol.

